# Comparative Analysis of Myofiber Characteristics, Shear Force, and Amino Acid Contents in Slow- and Fast-Growing Broilers

**DOI:** 10.3390/foods13243997

**Published:** 2024-12-11

**Authors:** Shuang Gu, Jia Gao, Zehao Li, Shenbo Zhang, Chaoliang Wen, Congjiao Sun, Wei Yan, Zhuocheng Hou, Ning Yang, Junying Li

**Affiliations:** 1 State Key Laboratory of Animal Biotech Breeding and Frontier Science Center for Molecular Design Breeding, China Agricultural University, Beijing 100193, China; 13592566171@163.com (S.G.); gaojia20161997@163.com (J.G.); lizehao1204@126.com (Z.L.); sy20243040890@cau.edu.cn (S.Z.); clwen@cau.edu.cn (C.W.); cjsun@cau.edu.cn (C.S.); wei.yan@cau.edu.cn (W.Y.); zchou@cau.edu.cn (Z.H.); nyang@cau.edu.cn (N.Y.); 2 National Engineering Laboratory for Animal Breeding and Key Laboratory of Animal Genetics, Breeding and Reproduction, Ministry of Agriculture and Rural Affairs, Department of Animal Genetics and Breeding, College of Animal Science and Technology, China Agricultural University, Beijing 100193, China; 3 Sanya Institute of China Agricultural University, Sanya 572025, China

**Keywords:** Wenchang chicken, Arbor Acres broiler, total number of myofibers, meat quality

## Abstract

Skeletal muscle fiber characteristics are pivotal in assessing meat quality. However, there is currently a lack of research precisely quantifying the total number of myofibers (TNM) of skeletal muscles. This study used Arbor Acres (AA) broilers and Wenchang (WC) chickens to determine the TNM of several skeletal muscles and the meat quality of the pectoralis major muscle (PM). The results showed that the TNMs of the PM in AA males and females were 935,363.64 ± 92,529.28 and 873,983.72 ± 84,511.28, respectively, significantly higher than those in WC (511,468.97 ± 73,460.81 and 475,371.93 ± 70,187.83) at 7 days of age (*p* < 0.01). In terms of gastrocnemius medialis in AA males and females, we recorded values of 207,551.43 ± 31,639.97 and 177,203.23 ± 28,764.01, showing a significant difference compared to the values observed in WC (146,313.03 ± 29,633.21 and 124,238.9 ± 20,136.95) (*p* < 0.01). Similarly, the levels of gastrocnemius lateralis exhibited a significant difference between AA and WC (*p* < 0.01). Furthermore, the essential, umami, and sweet amino acids were found to be significantly higher in WC compared to AA (*p* < 0.01). These findings offer valuable data and insights for accurately quantifying the TNM in livestock and for the development of further genetic breeding strategies for meat quality.

## 1. Introduction

Chicken meat consumption is increasing significantly around the world due to the benefits of high protein, low fat, and low calories offered by this meat [1,2,3]. After the intensive genetic selection for the rapid growth of broiler chickens over the past 70 years, the growth rate and meat yield of modern broilers have been improved markedly, and their body weight can reach up to ~3 kg during the 42-day rearing period [4,5]. In China, yellow-feathered chickens (slow-growing broilers), similar to white-feathered broilers (fast-growing broilers), are one of the most popular poultry farming species and meat resources [6]. The growth rate of yellow-feathered chickens is between that of layer chickens and white-feathered broilers, and the three types of yellow-feathered broilers are slow, medium, and fast-growing [6]. Wenchang (WC) chicken is a national, yellow-feathered chicken breed that is famous for its slow growth rate and excellent meat quality [7]. Arbor Acres (AA) broilers are a globally significant breed of fast-growing meat-type chickens that provide humans with an essential source of high-quality protein [8]. Chickens with different growth rates are often used as experimental material for studies related to development [9,10]. The rearing time of WC and AA broilers are 120 days and 42 days, respectively, making the exploration of differences in meat quality between the two breeds highly significant [11,12].

Skeletal muscle is the most abundant tissue in vertebrates, accounting for approximately 40% of body mass [13]. The fundamental functional units of skeletal muscle are multinucleated myofibers, which are formed by fusing mononucleated myoblasts during the embryonic development, and their number remains constant after birth [14]. The histological characteristics of myofibers have been found to be closely correlated with meat quality in previous studies [15,16]. The cross-sectional area (CSA) and density of myofibers are conventional indicators for assessing skeletal muscle development and are determined through the statistical analysis of partial images obtained from muscle tissue sections [17,18]. The total number of myofibers (TNM) is a value obtained through panoramic scanning of the myofibers in a specific muscle part, which directly reflects the muscle’s micro-morphological state [19]. Results produced by the method of estimating the TNM by multiplying the average fiber density—derived from several localized visual fields— by the cross-sectional area of the skeletal muscle have been found to differ from the actual count by approximately 25% [20]. In our previous studies, we used a large number of muscle tissue sections to develop an automatic detection tool for the TNM, named MyoV, which is based on deep learning techniques [20]. MyoV has achieved an accuracy of over 90%. This tool has significantly reduced the time required to determine the TNM and CSA of myofibers across various species and muscle tissues, providing an important method for investigating the intrinsic quality of muscle. The small size of the skeletal muscle in poultry before hatching can result in incomplete transverse sections in the majority of individuals during slide preparation, which may lead to the waste of both samples and valuable experimental time. The cross-section of skeletal muscle at D7 can be accommodated on a single slide, thereby preventing any potential underestimation of the fiber count [19]. Since the number of myofibers does not change after hatching, using the TNM determined at D7 as a valid indicator of the total myofiber count for the individual chicken is the appropriate method [19]. Meat tenderness is an important factor affecting meat quality, and it also influences consumers’ overall opinion [21]. Biochemical and functional analysis of meat has shown that lower shear force values were associated with greater tenderness [22]. Shear force values, measured perpendicular to the axis of myofibers, are closely related to the density of myofibers within the skeletal muscle [23]. Protein content is crucial for the integrity of myofibers, and, in turn, the density of myofibers influences the protein content of the skeletal muscle [24,25]. Furthermore, the concentration of amino acids in skeletal muscle significantly contributes to the flavor profile of meat [26]. Analysis of amino acids can determine the total content of amino acids in skeletal muscle, including the free amino acids present, as well as the amino acids included in proteins and peptides [27]. Skeletal muscle amino acid composition reflects the dynamic balance among protein synthesis, gluconeogenesis, and catabolism [28]. Humans require nine essential amino acids that are indispensable for our bodies and cannot be synthesized internally: histidine, isoleucine, leucine, lysine, methionine, phenylalanine, threonine, tryptophan, and valine [29]. Additionally, there are eleven non-essential amino acids that can be produced by the body but are still important for various functions: alanine, aspartic acid, asparagine, arginine, cysteine, glutamate, glutamine, glycine, proline, serine, and tyrosine. Most amino acids possess distinctive tastes, contributing to the multifaceted flavor profiles of foods [30]. Glutamic acid and aspartic acid are known for their savory taste, often referred to as umami. Alanine, glycine, serine, proline, and threonine are classified as sweet amino acids [31]. The content of essential amino acids and of umami and sweet amino acids in muscle tissue is critically important for muscle nutrition and palatability [32].

Although there have been numerous studies on the histological traits of muscle fibers in both fast-growing and slow-growing chicken breeds [33,34,35], no studies to date have specifically focused on analyzing the differences in the TNM in important skeletal muscle tissues. Consequently, this study employed the newly developed MyoV tool to compare the TNM in two specific chicken breeds to provide insights for poultry breeding.

## 2. Materials and Methods

### 2.1. Ethics Statement

The experimental procedures involving the chickens in this study strictly adhered to the protocols set forth by the Ministry of Science and Technology in Beijing, China. Ethical clearance was obtained from the Animal Welfare Committee of China Agricultural University, with the reference number AW71802202-1-2. This study was conducted following the established procedures detailed in the “Guide for the Care and Use of Laboratory Animals” (China Agricultural University).

### 2.2. Experimental Animal Handling and Sample Collection

The fertile eggs for AA broilers were sourced from Shandong Feng Xiang Group, while those for the WC chickens were procured from Wenchang City, Hainan Province, China. All eggs were disinfected using a Benzalkonium bromide solution before incubation. They were then incubated at a temperature of 37.8 °C and a relative humidity of 60% to facilitate hatching. After 21 days from hatching, 80 male and 80 female chicks from both the AA and WC groups were selected and raised uniformly at the Poultry Genetic Resources and Breeding Experimental Base of China Agricultural University, with ad libitum access to feed and water.

At 7 days of age (D7), 30 male and 30 female chicks from each group were fasted for 12 h before being humanely euthanized by cervical dislocation. The body weight (BW) as well as the weight of the pectoralis major muscle (PM) and the drumstick were carefully recorded for each bird. The left PM and the drumstick were harvested and fixed in 4% paraformaldehyde for more than 48 h. The remaining birds continued to be raised under the same conditions until they reached an age at which they were marketable. The AA broilers were raised until D42, and the WC chickens until D120. At this point, they were slaughtered. The BW, pectoralis major muscle weight (PMW), drumstick weight (DW), and gastrocnemius muscle weight (GW) of 30 males and 30 females per group were recorded. Their right PMs were bisected, and each of these were sealed in Ziplock bags of the same size at −20 °C until analysis.

### 2.3. Preperation of Muscle Paraffin Sections and H&E Staining

After fixation, the PM was excised and trimmed perpendicular to the direction of the myofiber to create a transverse section along the clavicle to the outer half of the pectoral muscle, which was then placed into an embedding cassette. Similarly, the drumstick was removed after fixation and sectioned at the maximum cross-sectional area to produce a transverse section. Subsequently, the embedding cassettes were dehydrated with gradient alcohol (75, 85, 90, 95%), cleared in xylene, and embedded in paraffin. The paraffin-embedded tissue blocks were cooled during the −20 °C freezing stage and then were cut into 4 µm sections. Following the spreading and baking processes, the sections were stained with hematoxylin and eosin (H&E) and sealed with neutral balsam. The prepared H&E-stained sections were then scanned using a slide scanner (3DHISTECH, Budapest, Hungary) to produce high-resolution, whole-slide images (WSIs).

### 2.4. Histological Analysis of Myofiber TNM and CSA

The WSIs of the PM, GM, and GL at D7 were first segmented into smaller image tiles of 1024 × 1024 pixels using MyoV. Subsequently, the image tiles were precisely quantified with the juvenile stage model of MyoV, which was designed for early developmental myofibers. The TNM of PM, GM, and GL for each individual at D7 was calculated by summing myofiber counts across all image tiles of a WSI for the two experimental groups. Among the extensive array of image tiles within a WSI, a representative subset of 50 tiles per individual was selected for CSA analysis, ensuring a uniform representation of muscle fiber distribution. The CSA of these selected tiles was evaluated using the aforementioned MyoV (v. 1.0) models. The average CSA of these 50 tiles was then computed to represent the individual’s mean CSA.

### 2.5. Determination of Shear Force and Amino Acid Content

The meat samples from 23 AA males and 25 AA females and from 27 WC males and 24 WC females were transferred from −20 °C to 4 °C for thawing. After this, the connective tissue and fat on the surface were removed from the meat samples. They were then placed in a 75 °C water bath pot in Ziplock bags and incubated until the internal temperature reached 75 °C; then, they were taken out of the bath pot. The PM samples were trimmed into a rectangular shape of 1 cm × 1 cm × 6 cm and then placed in a 4 °C refrigerator for 12 h to ensure thorough cooling. After cooling, the meat samples were subjected to shear force measurements. Specifically, the measurements were conducted using a meat quality analyzer (TA.XTplus, SMS, Godalming, UK) and applying a blade probe perpendicularly at three equidistant points along the length of each sample. The shear force was determined by averaging the three readings obtained from the anterior, central, and posterior regions of the meat sample. To compare the amino acid content differences between the two chicken breeds, 10 males of the PM of AA and WC at a marketable age were assessed for their content of 17 amino acids, including aspartic acid, threonine, serine, glutamic acid, glycine, alanine, cysteine, valine, methionine, isoleucine, leucine, tyrosine, phenylalanine, lysine, histidine, arginine, and proline. This analysis was conducted using an amino acid analyzer (L-8900, HITACHI, Tokyo, Japan) following the methods described by Li et al. [36].

### 2.6. Statistical Analyses

Statistical analysis was performed using R software (v. 4.2.3), with data expressed as mean ± SD. One- or two-way analysis of variance was applied to assess statistical differences between the experimental groups. A *p* value < 0.05 was considered statistically significant.

## 3. Results

### 3.1. Growth Performance Analysis

We firstly analyzed the impact of breed and sex on growth traits and muscle fiber histological characteristics at D7 and at a marketable age (Table 1). The results revealed that the breed had a more significant influence on the dependent variables than sex, and the interaction effects between breed and sex were essentially non-significant (Table 1). Thus, we focused on the differences in growth performance between the two breeds in subsequent analyses.

At D7, the BW of AA male chickens was 135.88 ± 14.37 g, which was more than double that of the WC male chickens (*p* < 0.01) (Table 2). The PMW of AA male chickens was 8.88 ± 1.26 g, nearly four times that of the WC male chickens (*p* < 0.01). The DW of AA male chickens was 10.48 ± 1.58 g, higher than the 5.06 ± 0.71 g of WC male chickens (*p* < 0.01). The growth performance differences between the females of two breeds at D7 were similar to those observed in the males. The commercial age of AA and WC was D42 and D120, respectively. The growth performance difference at the commercial age between AA and WC was compared (Table 3). The BW values of male and female AA chickens were 2611.63 ± 426.28 g and 2527.44 ± 245.65 g, respectively, which were higher than those of the WC chickens, with BW values of 2212.00 ± 200.02 g for males and 2180.89 ± 185.14 g for females (*p* < 0.01) (Table 3). Although there was considerable difference in skeletal muscle weights between the experimental groups, the PMW exhibited a greater disparity compared to the DW. The GW of AA and WC males was 64.21 ± 14.27 g and 62.27 ± 10.28 g, respectively, showing no significant difference. In contrast, a significant difference in GW was observed between AA and WC females (*p* < 0.01).

### 3.2. Myofiber Characteristics of Skeletal Muscle

**Figure 1:**
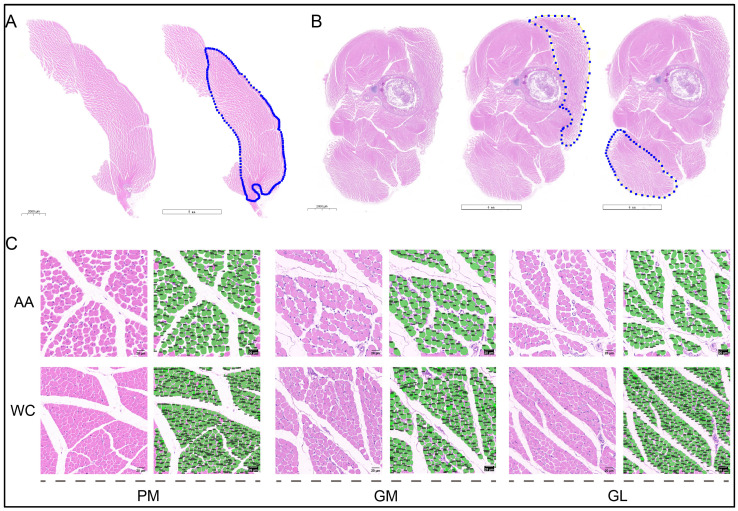
Skeletal muscle fibers of Arbor Acres (AA) broilers and Wenchang (WC) chickens at D7: (**A**) The prepared H&E-stained muscle sections were scanned to create whole slide images (WSIs). The left side displays an example of the WSIs for the pectoral muscle, and the right panel illustrates the region of interest, which corresponds to the pectoralis major muscle (PM) for subsequent sectioning and counting. (**B**) The left panel illustrates an example of WSI characteristics of the drumstick. The central and right panels represent the region of interest, corresponding to the gastrocnemius medialis (GM) and gastrocnemius lateralis (GL), respectively, with these areas clearly marked for further processing. (**C**) The first and second rows represent the image tiles of skeletal muscle sections from AA broilers and WC chickens, respectively, using males as representative examples. The images progress from left to right, depicting representative image tiles of the PM, the PM post-processed with MyoV software (v. 1.0), the GM, the GM enhanced through MyoV, the GL, and the GL after MyoV progressing. All images are presented at a resolution of 1024 × 1024 pixels, with a scale bar of 20 µm.

**Figure 2:**
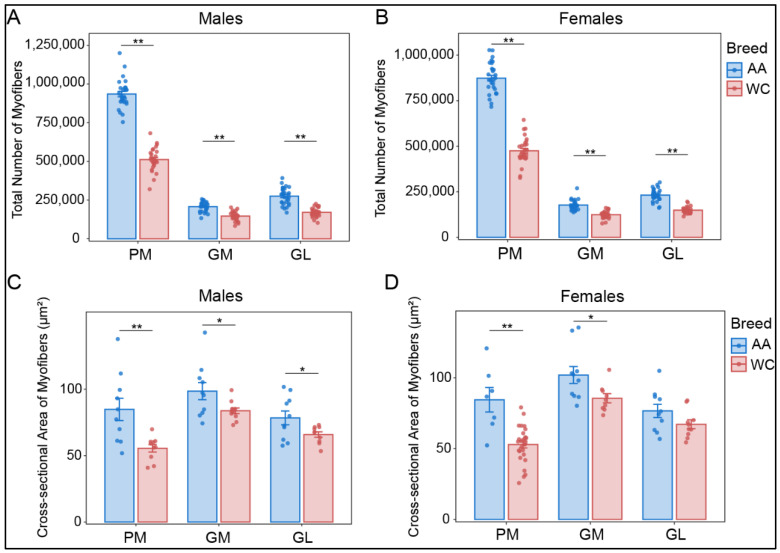
Muscle fiber characteristics of the two chicken breeds at D7: (**A**) The total number of myofibers (TNM) of the pectoralis major muscle (PM), gastrocnemius medialis (GM), and gastrocnemius lateralis (GL) of Arbor Acres (AA) broiler and Wenchang (WC) chicken males. (**B**) The TNM of PM, GM, and GL of AA and WC females. (**C**) The myofiber cross-sectional area (CSA) of different skeletal muscles of AA and WC males. (**D**) The CSA of PM, GM, and GL of AA and WC females. * indicates *p* < 0.05 and ** indicates *p* < 0.01.

The maximum cross-sectional area of the PM could be accommodated within a single slide due to the small body size of the chickens at D7, allowing for the precise determination of the TNM and CSA in the skeletal muscles of both AA and WC chicken breeds (Figure 1A,B). The WSIs of the PM, GM, and GL were segmented into image tiles of 1024 × 1024 pixels for the subsequent counting and processing of CSA (Figure 1C). The results showed that the TNM within the PM of AA males and females was higher than that in WC males and females (*p* < 0.01) (Table 2, Figure 2A,B). The TNM of the GM in WC males was also lower than that of AA (*p* < 0.01) (Table 2, Figure 2A). Similarly, the TNM of the GL in WC males was lower than that in AA males (*p* < 0.01) (Table 2, Figure 2A). The TNMs for the GM and GL in WC females were both lower than those in AA females (*p* < 0.01) (Table 2, Figure 2B).

Furthermore, comparative analysis was performed to assess the CSA of muscle fibers in the AA and WC breeds (Table 2). Specifically, the CSA of the PM in AA males was markedly higher than that observed in WC males (*p* < 0.01) (Figure 2C). The CSAs of the GM and the GL in AA males were higher than those in WC males (*p* < 0.05) (Figure 2C). The differences in muscle fiber CSA between AA and WC females were similar to those observed in males (Figure 2D).

### 3.3. Shear Force Difference Between Experimental Groups

The shear force of the PM in the AA group at D42 was higher than that in the WC group at D120. At D42, the shear force of AA males was 3670.43 ± 795.15 g, which was higher than that of the females, with a shear force of 3082.75 ± 664.66 g (*p* < 0.01) (Table 4). Conversely, at D120, a statistically significant disparity was noted in the shear force of the PM between WC roosters and hens, with respective values of 3340.29 ± 507.20 g and 2802.88 ± 396.34 g (*p* < 0.01) (Table 4).

### 3.4. Amino Acid Content Analysis

The amino acid content of the PM was determined for both AA and WC males, revealing that the total amino acid content in AA males was significantly lower, at 67.25 ± 3.80% compared to the 78.65 ± 0.86% in WC males (*p* < 0.01) (Table 5). Both the essential amino acids and the umami and sweet amino acids in WC males were found to be significantly higher than in AA males (*p* < 0.01). Specifically, among the essential amino acids, the level of lysine presented the highest value in the PM, with 7.34 ± 0.16% in WC males and 6.44 ± 0.42% in AA males. The content of lysine, leucine, valine, isoleucine, threonine, phenylalanine, and histidine in the PM was all higher in WC males than in AA males (*p* < 0.01). Moreover, the two umami amino acids and five sweet amino acids were also found in higher levels in WC males than in AA males (*p* < 0.01). Glutamic acid was found to be the most abundant, with 12.86 ± 0.17% in WC males and 10.42 ± 0.65% in AA males, followed by aspartic acid, with 7.72 ± 0.08% in WC males and 6.64 ± 0.38% in AA males. The content of sweet amino acids ranged between 2 and 5%.

## 4. Discussion

Slow- and fast-growing broilers are mainly prevalent in the China’s broiler industry [37]. Commercial fast-growing broiler chickens have superior production characteristics, including faster growth rates and greater feed efficiency compared to indigenous, slow-growing broiler chickens [38]. The slow-growing broiler chickens are small in body size, but they are delicious and possess suitable meat quality [25]. In this study, the differences in growth performance between AA and WC chickens are consistent with those found in previous research. The BW of AA chickens at D7 was approximately twice that of WC chickens, and the PMW was nearly four times that of WC chickens. Furthermore, the BW of WC chickens when developed at D120 was about 400 g less than that of AA chickens at D42, and the PMW of AA chickens was almost five times that of WC chickens. The disparity in the PMW between the two chicken breeds was significantly greater than the differences in the DW and GW, which were attributed to decades of intensive genetic selection on the carcass yield and breast muscle size of modern commercial broilers [39].

Mononuclear myoblasts fuse with each other to form small, multinucleated myotubes under the regulation of myogenic genes during the embryonic period [40,41]. The subsequent fusion of myoblasts with the multinucleated myotubes results in the formation of muscle fibers. In chickens, the initial establishment of muscle fiber morphology occurs at an embryonic age of 17 days [42]. It is notable that the number of myofibers in birds does not change after hatching, and muscle growth occurs as the myofibers hypertrophy [43,44]. The rate of muscle development is based on a certain number of muscle fibers formed during the embryonic period and the degree of muscle fiber hypertrophy post-hatching [45].

Thus, investigating the TNM in the skeletal muscles of fast-growing broilers and slow-growing broilers is crucial for subsequent research into livestock breeding. However, previous studies usually used a method for estimation that multiplied the average muscle fiber density by the muscle cross-sectional area [46,47]. The estimated TNM using this method can result in errors of about 25% compared to the ground truth [20]. This study precisely determined the TNM of several important skeletal muscles in fast-growing broilers (AA) and slow-growing broilers (WC) for the first time. The TNM in the PM of AA chickens was approximately 1.8 times that of WC chickens. The maximum cross-sectional area of the PM in modern broiler chickens is nearly four times larger than that of the PM in lines not subjected to selective breeding since the early 1950s [48]. Additionally, the differences in the TNM between AA and WC chickens in the GM and the GL was not as pronounced as those observed in the PM, which was consistent with the results of the DW and GW. This is likely due to the long-term intensive selection for the size and weight of modern broiler breast meat, which has resulted in greater differences in the PM between breeds compared to the differences observed in the gastrocnemius muscle [49]. The CSA of the skeletal muscles in AA chickens was larger than that in WC chickens; this finding was in accord with previous studies on the myofiber characteristics of slow- and fast- growing broilers [9,50].

Shear force serves as a crucial parameter in the evaluation of muscle quality. Recent studies have revealed that modern broiler breeds, such as Cobb 500 and AA, exhibit elevated shear forces in their muscle tissue in comparison to indigenous breeds such as the Beijing-You chicken, indicating a disparity in meat tenderness [51,52]. Consistent with these findings, our research demonstrates a similar pattern with the shear force of the PM in AA chickens surpassing that in WC chickens. This observation suggests that the muscle tenderness of WC broilers may outperform that of AA broilers, which could have implications for consumer preferences. Studies have indicated that certain dietary factors can influence meat shear force; for example, the addition of vitamin A to broiler diets can increase shear force [53], while feeding broilers with organic selenium sources has been shown to decrease this indicator [54]. From a genetic and breeding perspective, it is possible to develop broiler breeds that better meet consumer demands by selecting and breeding chickens with lower muscle shear force and superior tenderness.

Furthermore, amino acids, fatty acids, and nucleotides such as inosine are significant contributors to the taste and aroma of meat [55]. Previous research has shown that amino acids are correlated with the nutritional value and flavor quality of meats [56]. The PM of WC chickens contained higher levels of lysine and leucine compared to that of AA chickens. Chicken soup is a widely consumed food in Asian countries, as it is recognized for its capacity to deliver a comprehensive range of essential nutrients essential for human health while maintaining the nutritional integrity of chicken; these nutrients include free amino acids, polyunsaturated fatty acids, reducing sugars, and polysaccharides [57,58]. Amino acids play a significant role in imparting unique flavors such as sweetness, bitterness, and savoriness [59]. Umami amino acids are particularly important in chicken meat, with studies suggesting that higher concentrations of glutamate and aspartate may enhance the umami flavor of Mahuang chicken [38]. The metabolic pathways involving alanine, aspartate, and glutamate, as well as purine, glycine, serine, and threonine, are crucial in determining the aroma profile of chicken meat in Wuding chicken [60]. The results of our study have shown that WC chickens exhibit higher levels of sweet amino acids and lower shear force compared to AA chickens, which might suggest that the meat of WC chickens is more flavorful.

In summary, this study performs precise quantification of the TNM in different skeletal muscle tissues of slow- and fast-growing broilers for the first time. The TNM in the PM, GM, and GL of AA chickens was approximately 9 × 10^5^, 1.8 × 10^5^, and 2.5 × 10^5^, respectively, while the TNM of WC chickens was approximately 5 × 10^5^, 1.3 × 10^5^, and 1.5 × 10^5^, respectively. The CSA of AA chickens was larger than that of WC chickens, with the PM exhibiting greater intergroup discrepancies in CSA compared to the GM and GL. The shear force of AA chickens was found to be significantly higher than that of WC chickens, with males in both chicken breeds displaying greater shear force than females. Essential and umami and sweet amino acids, particularly lysine, leucine, aspartate, and glutamate, were more abundant and exhibited higher levels in WC chickens. This study shows that the small body size of WC chickens is linked to the lower TNM formed during embryonic development. As a local Chinese breed, the high quality of WC meat may be associated with its lower shear force and higher amino acid content. These findings provide valuable data for research on the TNM in poultry and offer significant insights for the genetic improvement in poultry meat quality traits through the control of histological characteristics. Future studies could explore TNM quantification in other species, as well as investigate how different environmental factors might affect muscle development.

## Figures and Tables

**Table 1:** Analysis of main effects and interaction effects of the factors.

Factors	BW	PMW	DW	GW	TNM of PM	TNM of GM	TNM of GL	CSA of PM	CSA of GM	CSA of GL
	D7 (*p*)									
Breed	0.000	0.000	0.000	-	0.000	0.000	0.000	0.000	0.002	0.009
Sex	0.070	0.000	0.044	-	0.000	0.000	0.000	0.788	0.582	0.956
Breed × Sex	0.697	0.009	0.850	-	0.399	0.434	0.106	0.829	0.865	0.694
	Marketable age (*p*)									
Breed	0.000	0.000	0.000	0.000	-	-	-	-	-	-
Sex	0.264	0.641	0.000	0.000	-	-	-	-	-	-
Breed × Sex	0.606	0.706	0.004	0.000	-	-	-	-	-	-

Abbreviations: D7: 7 days of age; BW = body weight; PMW = Pectoralis major muscle weight; DW = drumstick weight; GW = Gastrocnemius muscle weight; TNM = total number of myofibers; CSA = cross-sectional area; PM = Pectoralis major muscle; GM = Gastrocnemius medialis; and GL = Gastrocnemius lateralis.

**Table 2:** Growth traits and myofiber characteristics at D7.

Variables	Group	Males			Females		
		N	Mean ± SD	*p*	N	Mean ± SD	*p*
BW (g)	AA	30	135.88 ± 14.37 ^A^	0.000	30	131.40 ± 14.52 ^A^	0.000
	WC	30	69.97 ± 6.36 ^B^		30	67.07 ± 5.53 ^B^	
PMW (g)	AA	30	8.88 ± 1.26 ^A^	0.000	30	7.65 ± 1.41 ^A^	0.000
	WC	30	2.62 ± 0.48 ^B^		30	2.34 ± 0.37 ^B^	
DW (g)	AA	30	10.48 ± 1.58 ^A^	0.000	30	9.96 ± 1.76 ^A^	0.000
	WC	30	5.06 ± 0.71 ^B^		30	4.63 ± 0.58 ^B^	
TNM of PM	AA	28	935,363.64 ± 92,529.28 ^A^	0.000	29	873,983.72 ± 84,511.28 ^A^	0.000
	WC	29	511,468.97 ± 73,460.81 ^B^		30	475,371.93 ± 70,187.83 ^B^	
TNM of GM	AA	28	207,551.43 ± 31,639.97 ^A^	0.000	26	177,203.23 ± 28,764.01 ^A^	0.000
	WC	29	146,313.03 ± 29,633.21 ^B^		29	124,238.90 ± 20,136.95 ^B^	
TNM of GL	AA	30	275,639.20 ± 53,867.78 ^A^	0.000	27	231,897.04 ± 35,265.23 ^A^	0.000
	WC	31	170,538.06 ± 28,462.83 ^B^		29	148,809.03 ± 18,126.92 ^B^	
CSA of PM (µm^2^)	AA	10	84.80 ± 26.53 ^A^	0.004	7	84.52 ± 22.85 ^A^	0.000
	WC	10	55.49 ± 9.00 ^B^		28	52.96 ± 12.69 ^B^	
CSA of GM (µm^2^)	AA	10	98.52 ± 20.36 ^a^	0.036	10	102.01 ± 18.98 ^a^	0.030
	WC	11	83.76 ± 7.13 ^b^		9	85.60 ± 9.70 ^b^	
CSA of GL (µm^2^)	AA	10	78.44 ± 16.42 ^a^	0.037	10	76.65 ± 14.79	0.110
	WC	10	65.87 ± 6.41 ^b^		10	67.22 ± 9.90	

N: the number of observations per group. Abbreviations: D7: 7 days of age; BW = body weight; PMW = Pectoralis major muscle weight; DW = drumstick weight; TNM = total number of myofibers; CSA = cross-sectional area; PM = Pectoralis major muscle; GM = Gastrocnemius medialis; GL = Gastrocnemius lateralis; AA = Arbor Acres broilers; and WC = Wenchang chickens. Different capital letters indicate differences between groups at the *p* < 0.01 level, different lowercase letters indicate differences between groups at the *p* < 0.05 level, and no letters indicate no significant differences (*p* > 0.05).

**Table 3:** Growth performance at marketable age.

Variables	Group	Males			Females		
		N	Mean ± SD	*p*	N	Mean ± SD	*p*
BW (g)	AA_D42_	30	2611.63 ± 426.28 ^A^	0.000	30	2527.44 ± 245.65 ^A^	0.000
	WC_D120_	30	2212.00 ± 200.02 ^B^		30	2180.89 ± 185.14 ^B^	
PMW (g)	AA_D42_	30	413.78 ± 107.58 ^A^	0.000	30	403.79 ± 68.31 ^A^	0.000
	WC_D120_	30	97.99 ± 15.75 ^B^		30	96.93 ± 15.58 ^B^	
DW(g)	AA_D42_	30	265.86 ± 53.03 ^A^	0.000	30	243.03 ± 50.19 ^A^	0.000
	WC_D120_	30	216.20 ± 22.13 ^B^		30	151.58 ± 17.94 ^B^	
GW (g)	AA_D42_	30	64.21 ± 14.27	0.553	30	62.96 ± 8.83 ^A^	0.000
	WC_D120_	29	62.27 ± 10.28		30	41.71 ± 8.21 ^B^	

N: the number of observations per group. Abbreviations: BW = body weight; PMW = Pectoralis major muscle weight; DW = drumstick weight; GW = Gastrocnemius muscle weight; AA_D42_ = Arbor Acres broilers at D42; and WC_D120_ = Wenchang chickens at D120. Different capital letters indicate differences between groups at the *p* < 0.01 level, and no letters indicate no significant differences (*p* > 0.05).

**Table 4:** Shear force of PM at marketable age.

Group	N	Mean ± SD	*p*	N	Mean ± SD	*p*
	AA_D42_			WC_D120_		
Males	23	3670.43 ± 795.15	0.008	27	3340.29 ± 507.20	0.000
Females	25	3082.75 ± 664.66		24	2802.88 ± 396.34	
	Males			Females		
AA_D42_	23	3670.43 ± 795.15	0.082	25	3082.75 ± 664.66	0.081
WC_D120_	27	3340.29 ± 507.20		24	2802.88 ± 396.34	

N: the number of observations per group. The unit of shear force is g. Abbreviations: PM = Pectoralis major muscle; AA_D42_ = Arbor Acres broilers at D42; and WC_D120_ = Wenchang chickens at D120.

**Table 5:** Amino acid content in PM of males at marketable age.

Content, %	Group	N	Mean ± SD	CV, %	Minimum	Maximum
**Total amino acids**	AA_D42_	10	67.25 ± 3.80 ^B^	5.65	59.19	70.77
	WC_D120_	10	78.65 ± 0.86 ^A^	1.09	76.87	79.93
**Essential amino acids**	AA_D42_	10	29.99 ± 1.72 ^B^	5.74	26.50	31.77
	WC_D120_	10	34.76 ± 0.45 ^A^	1.29	33.97	35.47
Lysin	AA_D42_	10	6.44 ± 0.42 ^B^	6.52	5.59	6.86
	WC_D120_	10	7.34 ± 0.16 ^A^	2.18	7.01	7.50
Leucine	AA_D42_	10	5.66 ± 0.32 ^B^	5.65	4.98	5.97
	WC_D120_	10	6.68 ± 0.06 ^A^	0.90	6.55	6.76
Valine	AA_D42_	10	3.79 ± 0.21 ^B^	5.54	3.39	3.99
	WC_D120_	10	4.33 ± 0.05 ^A^	1.15	4.22	4.40
Isoleucine	AA_D42_	10	3.58 ± 0.20 ^B^	5.59	3.17	3.79
	WC_D120_	10	4.11 ± 0.04 ^A^	0.97	4.03	4.16
Threonine	AA_D42_	10	3.12 ± 0.17 ^B^	5.45	2.76	3.31
	WC_D120_	10	3.67 ± 0.05 ^A^	1.36	3.58	3.75
Phenylalanine	AA_D42_	10	2.72 ± 0.14 ^B^	5.15	2.44	2.87
	WC_D120_	10	3.24 ± 0.05 ^A^	1.54	3.17	3.31
Histidine	AA_D42_	10	2.48 ± 0.16 ^B^	6.45	2.24	2.77
	WC_D120_	10	3.18 ± 0.21 ^A^	6.60	2.83	3.51
Methionine	AA_D42_	10	2.21 ± 0.15	6.79	1.92	2.36
	WC_D120_	10	2.21 ± 0.03	1.36	2.17	2.25
**Umami amino acids**	AA_D42_	10	17.06 ± 1.03 ^B^	6.04	14.83	18.21
	WC_D120_	10	20.58 ± 0.24 ^A^	1.17	20.13	20.91
Glutamate	AA_D42_	10	10.42 ± 0.65 ^B^	6.24	8.97	11.16
	WC_D120_	10	12.86 ± 0.17 ^A^	1.32	12.58	13.08
Aspartic acid	AA_D42_	10	6.64 ± 0.38 ^B^	5.72	5.86	7.05
	WC_D120_	10	7.72 ± 0.08 ^A^	1.04	7.56	7.83
**Sweet amino acids**	AA_D42_	10	15.79 ± 0.80 ^B^	5.07	14.05	16.52
	WC_D120_	10	18.34 ± 0.21 ^A^	1.15	17.86	18.60
Alanine	AA_D42_	10	4.27 ± 0.25 ^B^	5.85	3.74	4.49
	WC_D120_	10	4.89 ± 0.05 ^A^	1.02	4.77	4.96
Glycine	AA_D42_	10	3.09 ± 0.15 ^B^	4.85	2.76	3.24
	WC_D120_	10	3.72 ± 0.06 ^A^	1.61	3.63	3.84
Threonine	AA_D42_	10	3.12 ± 0.17 ^B^	5.45	2.76	3.31
	WC_D120_	10	3.67 ± 0.05 ^A^	1.36	3.58	3.75
Serine	AA_D42_	10	2.61 ± 0.12 ^B^	4.60	2.35	2.75
	WC_D120_	10	3.13 ± 0.05 ^A^	1.60	3.02	3.20
Proline	AA_D42_	10	2.70 ± 0.14 ^B^	5.19	2.43	2.83
	WC_D120_	10	2.94 ± 0.04 ^A^	1.36	2.86	2.98

N: the number of observations per group. Abbreviations: AA_D42_ = Arbor Acres broilers at D42; and WC_D120_ = Wenchang chickens at D120. Different capital letters indicate differences between groups at the *p* < 0.01 level, and no letters indicate no significant differences (*p* > 0.05).

## Data Availability

The original contributions presented in this study are included in the article, and further inquiries can be directed to the corresponding author.

## Abbreviations

WC	Wenchang chickens;
CSA	Cross-sectional area;
TNM	Total number of myofibers;
AA	Arbor Acres broilers;
D7	7 days of age;
BW	Body weight;
DW	Drumstick weight;
PM	Pectoralis major muscle;
PMW	Pectoralis major muscle weight;
GW	Gastrocnemius muscle weight;
GL	Gastrocnemius lateralis;
GM	Gastrocnemius medialis;
H&E	Hematoxylin and eosin;
WSIs	Whole-slide images.
